# Genetic variants in regulatory regions of microRNAs are associated with lung cancer risk

**DOI:** 10.18632/oncotarget.10299

**Published:** 2016-06-25

**Authors:** Kaipeng Xie, Cheng Wang, Na Qin, Jianshui Yang, Meng Zhu, Juncheng Dai, Guangfu Jin, Hongbing Shen, Hongxia Ma, Zhibin Hu

**Affiliations:** ^1^ Department of Epidemiology and Biostatistics, Collaborative Innovation Center of Cancer Medicine, School of Public Health, Nanjing Medical University, Nanjing, 211166, China; ^2^ Jiangsu Key Laboratory of Cancer Biomarkers, Prevention and Treatment, School of Public Health, Nanjing Medical University, Nanjing, 211166, China

**Keywords:** non-small cell lung cancer, microRNA, susceptibility, survival

## Abstract

Genetic variants in regulatory regions of some miRNAs might be associated with lung cancer risk and survival. We performed a case-control study including 1341 non-small cell lung cancer (NSCLC) cases and 1982 controls to evaluate the associations of 7 potentially functional polymorphisms in several differently expressed miRNAs with NSCLC risk. Each SNP was also tested for the association with overall survival of 1001 NSCLC patients. We identified that rs9660710 in miR-200b/200a/429 cluster and rs763354 in miR-30a were significantly associated with NSCLC risk [odds ratio (OR) = 1.17, 95% confidence interval (CI) = 1.06–1.30, *P* = 0.002; OR = 0.88, 95% CI = 0.80–0.98, *P* = 0.017; respectively]. However, no significant association between variants and NSCLC death risk was observed in survival analysis. Functional annotation showed that both rs9660710 and rs763354 were located in regulatory elements in lung cancer cells. Compared to normal tissues, miR-200a-3p, miR-200a-5p, miR-200b-3p, miR-200b-5p and miR-429 were significantly increased in The Cancer Genome Atlas (TCGA) Lung Adenocarcinoma (LUAD) tumors, whereas miR-30a-3p and miR-30a-5p were significantly decreased in tumors (all *P* < 0.05). Furthermore, we observed that rs9660710 is an expression quantitative trait locus (eQTL) or methylation eQTL for miR-429 expression in TCGA normal tissues. Our results indicated that rs9660710 in miR-200b/200a/429 cluster and rs763354 in miR-30a might modify the susceptibility to NSCLC.

## INTRODUCTION

Non-small cell lung cancer (NSCLC) accounts for over 80% of lung cancer, which remains the leading cause of cancer-related death worldwide [1, 2]. Although tobacco consumption is a major risk factor for lung cancer, accumulating evidence suggests that genetic variations may play a vital role in tumor development and progression [3–6]. Therefore, identification of these genetic biomarkers may enhance risk prediction and improve clinical outcomes of NSCLC patients.

Emerging evidence has suggested that some key microRNAs (miRNAs) are consistently dysregulated in lung cancer tissues and function as oncogenes or tumor suppressor genes in the initiation and progression of lung cancer [7–10]. Notably, Guan *et al.* synthesized fourteen microarray-based human lung cancer miRNA expression profiling studies including a total of 585 tumor and 519 non-cancerous control samples, and employed a vote-counting strategy to identify several consistent differentially miRNAs, such as up-regulated miR-210, miR-21, miR-183, miR-182, miR-200b, and down-regulated miR-30a, miR-145, miR-143 and miR-451a [8]. Furthermore, using a meta-analysis with more than 1100 lung cancer and noncancerous samples from 20 original studies, another study also confirmed these abnormally expressed miRNAs [9]. Meanwhile, previous studies have suggested that the high expression of miR-210, miR-21, miR-183, miR-182, and low expression of miR-30a, miR-145, miR-143 were associated with poor prognosis of lung cancer [11–16]. These studies provided the promising candidates for further researches on miRNAs in lung cancer development and progression.

Interestingly, the biological function of these miRNAs has also been explored in lung cancer. For example, miR-21 located in the region of 17q23.1 promotes lung tumorigenesis through inhibition of negative regulators of the Ras/MEK/ERK pathway and apoptosis [17]. MiR-210 localizes in chromosome 11p15.5 and acts as an important regulator of the cellular response to hypoxia [18]. MiR-182 and miR-183 belong to the members of the miR-183/96/182 cluster, the overexpression of which has been identified as a potential risk factor for lung cancer development [19]. MiR-200b/200a/429 clustered at chromosome 1p36 and miR-30a located at 6q13 are key regulators of epithelial-mesenchymal transition (EMT) and thus affect the cell migration, invasion and metastasis in lung cancer [20–22]. MiR-451a inhibits ras-related protein 14 (*RAB14*) expression and functions as a tumor suppressor in human non-small cell lung cancer [10]. MiR-145 and miR-143 located in 5q33.1 inhibit NSCLC cell growth and metastasis by targeting epidermal growth factor receptor (*EGFR*) and nucleoside diphosphate linked moiety X-type motif 1 (*NUDT1*) or LIM domain kinase 1 (*Limk1*) [23, 24]. Taken together, these miRNAs have been identified to play the important role in the NSCLC tumorigenesis and progression.

In recent years, comprehensive analyses based on genome-wide association studies (GWAS) have indicated that disease/trait-associated loci are enriched in the genomic regulatory regions [25–27]. Thus, it is biologically plausible that single nucleotide polymorphisms (SNPs) in the regulatory regions of miRNA may also contribute to phenotypic variations in the human population [25]. Therefore, we evaluate the effect of potentially functional polymorphisms within the 10 kb upstream region of the above-mentioned key miRNAs cluster or single miRNA (miR-143/145, miR-183/96/182 cluster, miR-21, miR-210, miR-200b/200a/429 cluster, miR-30a, miR-451a) on the NSCLC risk and survival in a Chinese Han population.

## RESULTS

### Subjects characteristics

Characteristics of all subjects (1341 cases and 1982 controls) have been described in Supplementary Table S1. The age and gender were comparable between cases and controls (*P*_age_ = 0.972, *P*_gender_ = 0.179). The cases have a higher rate of smoking (61.1%) compared with the controls (48.5%), and most cases (64.1%) are diagnosed with adenocarcinoma.

### Associations of 7 SNPs with NSCLC risk

Based on the bioinformatics methods (see Materials and Methods), 7 potentially functional SNPs (rs3733846 in miR-143/145, rs12538588 in miR-183/96/182, rs1292060 in miR-21, rs763354 in miR-30a, rs9660710 in miR-200b/200a/429, rs12286521 in miR-210 and rs901975 in miR-451a) were genotyped in our study. The genotype distributions of these SNPs between cases and controls are shown in Table 1. After adjusting for age, gender, and smoking, the minor allele of rs9660710 (C > A) in miR-200b/miR200a/429 demonstrated a significant association with an increased risk of NSCLC [additive model: Odds ratios (OR) = 1.17, 95% confidence intervals (CI) = 1.06–1.30, *P* = 0.002], while the variant allele of rs763354 (G > A) in miR-30a was associated with a decreased risk (additive model: adjusted OR = 0.88, 95%CI = 0.80–0.98, *P* = 0.017). No significant association was observed between other five SNPs and NSCLC risk.

**Table 1:** Primary information of 7 potentially functional SNPs and associations with NSCLC risk

miRNAs	SNP	Allele^a^	Cases^b^	Controls^b^	MAF^c^	Adjusted OR (95%CI)^d^				*P*^f^
						Additive model	Dominant model	Codominant model^e^		
								het	hom	
miR-143/145	rs3733846	A/G	580/597/164	839/925/218	0.34/0.34	1.01 (0.91–1.13)	0.97 (0.84–1.11)	0.93 (0.80–1.08)	1.11 (0.88–1.40)	0.810
miR-183/96/182	rs12538588	G/A	1229/112/0	1818/163/0	0.04/0.04	0.98 (0.76–1.26)	0.98 (0.76–1.26)	—	—	0.874
miR-21	rs1292060	A/G	435/645/261	648/928/397	0.44/0.44	1.00 (0.90–1.10)	1.01 (0.87–1.18)	1.03 (0.87–1.20)	0.99 (0.81–1.21)	0.972
miR-30a	rs763354	G/A	486/640/210	650/965/352	0.40/0.42	**0.88** **(0.80–0.98)**	**0.85** **(0.74–0.99)**	0.88 (0.75–1.03)	**0.78** **(0.64–0.97)**	**0.017**
miR-200b/200a/429	rs9660710	C/A	431/669/240	746/930/306	0.43/0.39	**1.17** **(1.06–1.30)**	**1.26** **(1.09–1.46)**	**1.23** **(1.05–1.44)**	**1.34** **(1.09–1.65)**	**0.002**
miR-210	rs12286521	A/G	752/510/79	1126/738/118	0.25/0.25	1.02 (0.91–1.14)	1.03 (0.90–1.19)	1.04 (0.90–1.21)	0.98 (0.72–1.32)	0.792
miR-451a	rs901975	G/A	571/601/169	861/873/245	0.35/0.34	1.03 (0.93–1.14)	1.04 (0.90–1.20)	1.04 (0.90–1.21)	1.04 (0.83–1.30)	0.627

a Major/minor allele.

b Major homozygote/heterozygote/minor homozygote in cases and controls.

c Minor allele frequency among cases/controls.

d Logistic regression with adjustment for age, gender and smoking.

e het: heterozygote versus major homozygote; hom: minor homozygote versus major homozygote.

f *P* for additive model.

Bold values are statistically significant.

Furthermore, we performed stratification analysis on the associations of rs9660710 and rs763354 with NSCLC risk by age, gender, smoking and histological subtype (Table 2). The heterogeneity test showed that the difference was significant in the subgroup of age for rs763354 (*P* = 0.040), and the protective effect of rs763354 A allele in subjects aged ≥ 60 years (adjusted OR = 0.80, 95%CI = 0.70–0.92, *P* = 0.002) was prominent as compared with subjects aged < 60 years (adjusted OR = 0.99, 95%CI = 0.85–1.15, *P* = 0.847).

**Table 2:** Stratification analysis of rs9660710 and rs763354 genotypes associated with NSCLC risk

Variables	rs9660710 (CC/CA/AA)^a^		OR (95% CI)^b^	*P*^c^	rs763354 (GG/GA/AA)^a^		OR (95% CI)^b^	*P*^c^
	Cases	Controls			Cases	Controls		
Age (years)								
< 60	187/293/109	334/397/142	**1.18** **(1.01–1.37)**	1.000	210/276/100	309/416/147	0.99 (0.85–1.15)	**0.040**
≤ 60	244/376/131	412/533/164	**1.18** **(1.02–1.35)**		276/364/110	341/549/205	**0.80** **(0.70–0.92)**	
Gender								
Male	310/477/161	504/649/205	**1.13** **(1.00–1.27)**	0.296	357/439/149	446/677/233	**0.86** **(0.76–0.97**)	0.443
Female	121/192/79	242/281/101	**1.27** **(1.06–1.53)**		129/201/61	204/288/119	0.94 (0.77–1.13)	
Smoking								
Never	175/254/93	389/469/162	1.14 (0.98–1.33)	0.624	186/251/85	342/492/175	0.95 (0.81–1.11)	0.256
Ever	256/415/147	357/461/144	**1.20** **(1.05–1.38)**		300/389/125	308/473/177	**0.84** **(0.73–0.97)**	
Histological types								
Squamous cell carcinoma	151/231/99	746/930/306	**1.28** **(1.10–1.49)**	0.206	172/234/73	650/965/352	0.86 (0.74–1.00)	0.725
Adenocarcinoma	280/438/141	746/930/306	**1.13** **(1.00–1.27)**		314/406/137	650/965/352	**0.89** **(0.79–1.00)**	

a Major homozygote/heterozygote/minor homozygote.

b Adjusted for age, gender and smoking where appropriate in additive model.

c *P* values were from heterogeneity test based on *χ*^2^-based *Q* test.

Bold values are statistically significant.

### Relationships between 7 SNPs and NSCLC survival

We further investigated the relationships between these SNPs and NSCLC survival. The clinical characteristics of 1001 NSCLC patients are described in Supplementary Table S2. We found that some factors, including gender, smoking, surgery, clinical stage and chemotherapy or radiotherapy had significant influences on patient prognosis (log-rank *P* < 0.05). Associations of these SNPs with overall survival in different genetic models are shown in Supplementary Table S3. Log-rank test indicated that no variant showed a significant association with overall survival time in additive, dominant or co-dominant models (all *P* > 0.05). The results were similar after adjusting for age, gender, smoking, clinical stage, chemotherapy or radiotherapy, surgery status and histological types (all *P* > 0.05, Supplementary Table S4).

### *In silico* analysis of promising variants and miRNAs

**Figure 1:**
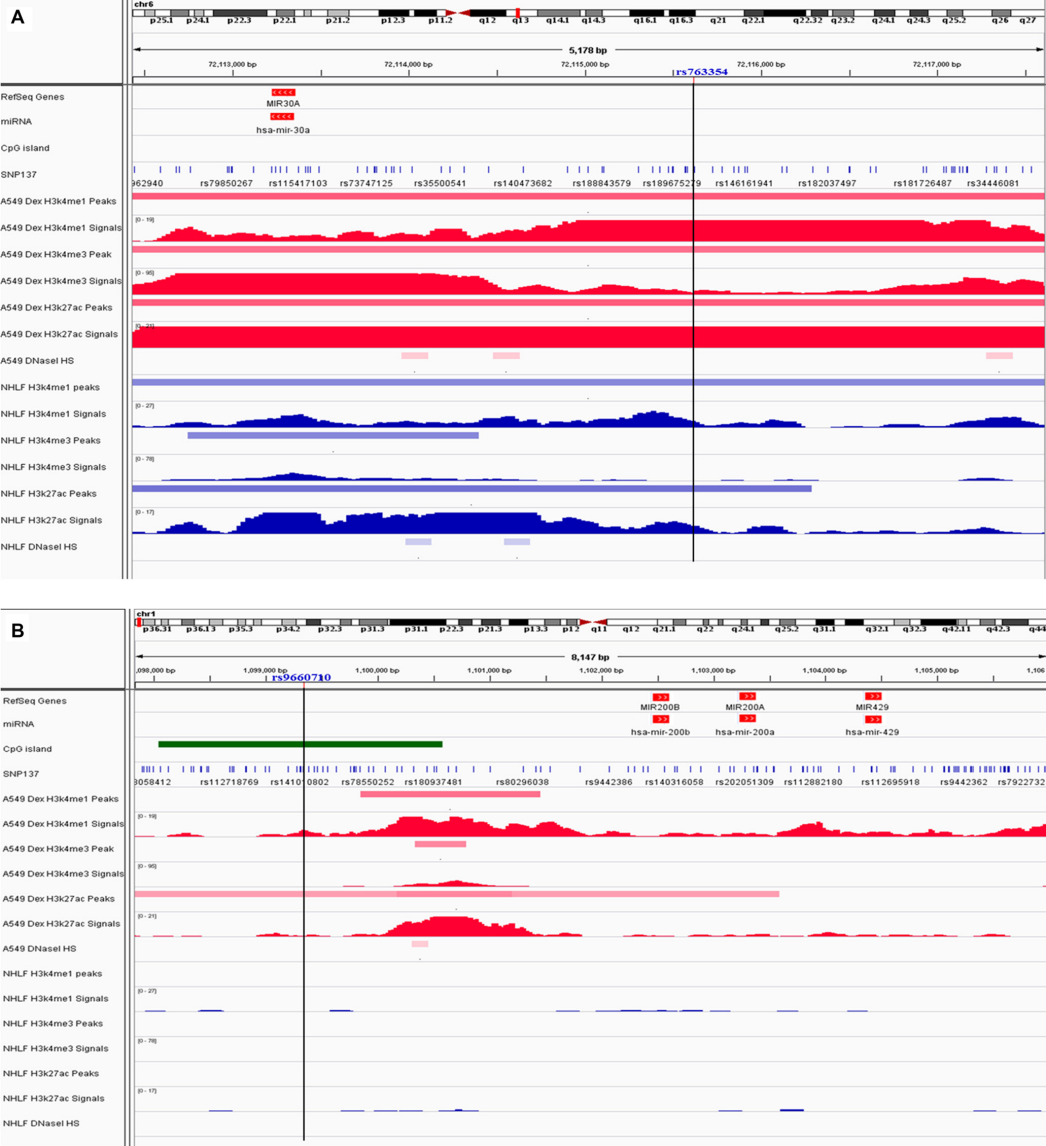
Chromatin features of risk-associated SNPs Functional annotation in proximity to miR-30a rs763354 location (**A**) and miR-200b/200a/429 cluster rs9660710 location (**B**) in A549 and NHLF cells. ChIP-seq tracks for promoter histone marks (H3K4me3) and enhancer histone marks (H3K4me1, H3K27ac) in A549 and NHLF cells are present along with DNAse hypersensitivity tracks from ENCODE data. The black vertical lines guide the position of risk-associated SNPs. The blue stripe represents the normal lung fibroblasts (NHLF). The red stripe represents the lung cancer cell line (A549). The green stripe represents the CpG island in the region.

We annotated two significantly risk-associated variants (rs763354, rs9660710) in regulatory elements cataloged in Encyclopedia of DNA Elements (ENCODE) project (https://www.encodeproject.org/), and HaploReg (http://compbio.mit.edu/HaploReg), respectively. Visual inspection of the SNPs and histone modification peaks showed that rs763354 is situated within the enhancer element (H3K4me1and H3K27ac histone mark) in both normal lung cell line (Normal Human Lung Fibroblasts, NHLF) and lung cancer cell line (A549), and fall into the promoter element (H3K4me3) in A549 cell line (Figure 1A). Rs9660710 is situated in the enhancer element (H3K27ac) in A549 cell line (Figure 1B). Furthermore, based on the HaploReg data, we found that rs763354, rs9660710 and their correlated variants within a LD block are positioned in regulatory regions (histone promoter or enhancer marks in several tissues), and alter multiple regulatory motifs or disturb some proteins bound (Supplementary Tables S5–S6). Taken together, our results indicated that these SNPs were probably involved in the regulation of miRNA expression.

**Figure 2:**
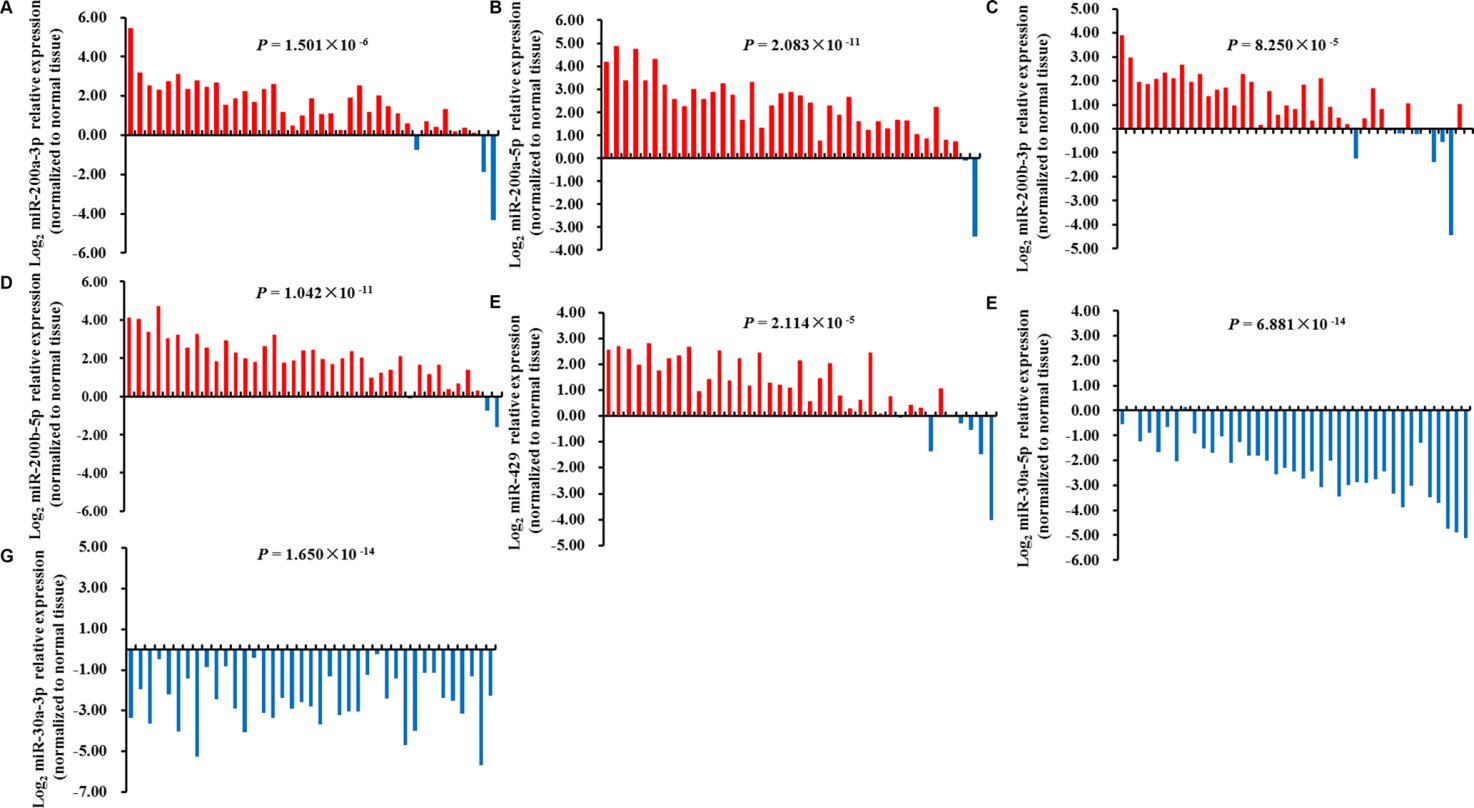
Expression levels of candidate miRNAs in 39 paired TCGA LUAD tissues miR-200a-3p (**A**), miR-200a-5p (**B**), miR-200b-3p (**C**), miR-200b-5p (**D**), miR-429 (**E**) were significantly increased in tumor tissues in comparison with paired normal tissues, while miR-30a-5p (**F**), miR-30a-3p (**G**) were significantly decreased in tumor tissues. The statistical analysis was performed using paired student's *t*-test.

To further explore the influence of rs763354 and rs9660710 on their related miRNAs (miR-30a-3p, miR-30-5p, miR-200a-3p, miR-200a-5p, miR-200b-3p, miR-200b-5p and miR-429) in NSCLC development, we used publicly available TCGA Lung Adenocarcinoma (LUAD) datasets and firstly evaluated the association of rs763354 and rs9660710 genotypes with their mature miRNAs expression in 38 adjacent normal tissues. We observed that rs9660710 (C > A) was positively associated with miR-429 expression (rho = 0.388, *P* = 0.016), indicating that rs9660710 may be an expression quantitative trait locus (eQTL) for miR-429. However, no significant association was observed between this SNP and the expression of miR-200b (*P*_miR-200b-3p_ = 0.059, *P*_miR-200b-5p_ = 0.190) and miR-200a (*P*_miR-200a-3p_ = 0.093, *P*_miR-200a-5p_ = 0.120). Additionally, there was no significant association between rs763354 and miR-30a expression (*P*_miR-30a-5p_ = 0.833, *P*_miR-30a-3p_ = 0.648). Subsequently, we evaluated these miRNA expression levels in 39 paired tissues. Consistent with previous studies, miR-30a-3p and miR-30a-5p were significantly decreased in tumor tissues in comparison with paired normal tissues while others (miR-200a-3p, miR-200a-5p, miR-200b-3p, miR-200b-5p and miR-429) were significantly increased in tumor tissues (Figure 2). Taken together, the minor allele of rs9660710 may increase the miR-429 expression and thus contribute to lung tumorigenesis.

Interestingly, rs9660710 (C > A) located in the CpG island in the promoter of miR-200b/200a/429 can lead to the loss of the CpG site (Figure 1B), which may prevent the CpG site from being methylated and increasing the miR-429 expression. To support the hypothesis, we extracted the TCGA LUAD DNA methylation data, which covers 51 CpG sites in the 10kb 5′ upstream region of miR-200b/200a/429 cluster. We observed that rs9660710 was negatively associated with the methylation level of cg00152708 in 32 normal tissues (rho = −0.427, *P* = 0.017). Meanwhile, the methylation level of cg00152708 was significantly lower in 29 tumor tissues compared to the adjacent normal tissues (0.457± 0.135 vs 0.553 ± 0.063, *P* = 0.003). However, due to the limited normal tissues (6 samples) with both DNA methylation and miRNA expression information, we cannot directly analyze the relationship between the methylation level of cg00152708 and miR-429 expression. Therefore, we speculated that rs9660710 (C > A) may decrease cg00152708 methylation level and thus increase the miR-429 expression in lung carcinogenesis, indicating a role of rs9660710 as a methylation quantitative trait locus (meQTL) for miR-429 expression.

## DISCUSSION

In the present study, we explored the associations of 7 SNPs in regulatory regions of candidate miRNAs with the NSCLC risk and survival. Our findings suggested that rs9660710 in miR-200b/200a/429 cluster was associated with an increased NSCLC risk, and miR-30a rs763354 was associated with a decreased NSCLC risk. However, no variant was associated with NSCLC prognosis.

Previous studies demonstrated that abnormal expression of miR-200b/200a/429 cluster and miR-30a could contribute to lung tumorigenesis, and may be potential diagnostic biomarkers for NSCLC. For example, one recent study reported that upregulation of miR-200 could active several key components of the PI3K pathway to promoter lung cancer cells growth and contribute to early lung tumorigenesis [28]. Lang *et al.* observed that the expression of miR-429 increased in primary NSCLS tissues and cell lines, and overexpression of miR-429 promoted NSCLC cells proliferation through inhibiting *PTEN*, *RASSF8* and *TIMP2* expression [29]. For miR-30a, its overexpression was indicated to suppress NSCLC cell proliferation and trigger cell cycle arrest by inhibiting cell cycle regulators (*CDK2*, *CDK4*, *Cyclin A2*, *Cyclin D1*) [30]. Consistent with these previous findings, we found that miR-200a-3p, miR-200a-5p, miR-200b-3p, miR-200b-5p and miR-429 in our study were significantly overexpressed in tumors compared with adjacent normal tissues in TCGA LUAD data, whereas the expression levels of miR-30a-3p and miR-30a-5p were suppressed in tumors.

To date, only few studies have focused on the associations of above miRNAs polymorphisms with cancer development and prognosis. Leng *et al.* identified the G allele of rs61768479, a tagSNP located in the promoter region of miR-200a/200b/429, was associated with a 50% reduced risk of lung cancer in Caucasians; however, this association was not confirmed in the validation stage [31]. Wu *et al.* reported that rs1045385 in the 3′ UTR of AP-2α suppressed the binding of miR-200b/200c/429 to AP-2α, and might be a potential prognostic marker for cisplatin treatment [32]. Another study showed that miR-143/145 rs3733845 confers protective effect on the risk of colorectal cancer in Chinese population [33]. In the present study, we failed to analyze the associations between these two SNPs (rs61768479 and rs1045385) and NSCLC risk and survival, owing to the low frequency in Chinese Han population and the reason that rs1045385 is located in the target gene. But for rs3733845, we did not observed the significant association of this SNP with lung cancer risk in our study, which may be due to the complex effects of polymorphisms on different cancer types. Our study showed for the first time that rs9660710 in miR-200b/200a/429 cluster conferred the increased risk of NSCLC in Chinese population, but miR-30a rs763354 showed a protective effect on NSCLC risk.

*In silico* analysis suggested that rs763354 and rs9660710 were situated in different histone modification patterns between normal and lung cancer cells, highlighting the possibility that these loci may regulate the transcription of miRNAs. Functional annotations from HaploReg indicated that rs763354 might increase the binding of *Sox2*, which might bind to the promoter regions of miRNAs [34]. However, we did not observe significant association between rs763354 and miR-30a-3p or miR-30a-5p expression in 38 TCGA adjacent normal tissues. The variant allele of rs9660710 (C > A) is predicted to strengthen the affinity motifs for *BRCA1* (Supplementary Table S5), which participates in transcriptional regulation by interacting with a large amount of different transcription factors, including *TP53* and *CMYC* [35]. In our study, we observed that the variant allele of rs9660710 is an eQTL for miR-429 expression and significantly increase the miR-429 expression in normal tissues. Moreover, rs9660710 is a CpG site related SNP (cgSNP) [36] and meQTL for miR-429 expression. Consequently, we speculated that rs9660710 may disrupt transcription factor response elements or DNA methylation to promote the expression of miR-429, respectively. However, further functional characterizations are warranted to validate the genotype-phenotype correlation.

In conclusion, our study provided evidence that SNPs in miRNAs regulatory regions were significantly associated with NSCLC risk, indicating the importance of the functional variants of miRNAs in NSCLC tumorigenesis. Larger studies with functional investigations are needed to validate the findings.

## MATERIALS AND METHODS

### Study subjects

All subjects in this study have been described previously [37]. In brief, 1341 patients with NSCLC were newly diagnosed and recruited from the First Affiliated Hospital of Nanjing Medical University and the Cancer Hospital of Jiangsu Province since 2003. All patients were histopathologically or cytologically confirmed NSCLC. The 1982 controls were selected from healthy individuals participated in screening for non-infectious diseases during the same time period. The controls were frequency-matched to cases on age and gender. After having signed the informed consent, each participant was face-to-face interviewed by trained interviewers to collect personal information on demographics data. Furthermore, we collected patients' clinical information from patients' medical records and subsequent personal or family contacts, including the date of diagnosis, surgery status, clinical stage, chemotherapy or radiotherapy, histological types and the date of death during follow-up (last follow-up in August 2013). Finally, a total of 1001 cases (74.6%) had sufficient information about survival time to permit statistical analysis with the median survival time (MST) of 26.0 months. This study was approved by the institutional review board of Nanjing Medical University.

### SNPs selection and genotyping

Based on the UCSC Genome Browser database (http://genome.ucsc.edu/) and HapMap SNP database (phase II + III Feb 09, on NCBI B36 assembly, dbSNP b126), 38 common SNPs (MAF ≥ 0.05 in Chinese Han population) were selected in the 10kb 5′ upstream region of each pre-miRNA. If the candidate miRNA is located in a cluster, we then screened the upstream region of the cluster instead of the single miRNA (miR-143/145, miR-183/96/183, miR-21, miR-210, miR-200b/200a/429, miR-30a, miR-451a). The potentially functional SNPs were predicted *in silico* by using the SNPinfo web server (http://snpinfo.niehs.nih.gov/). Among 16 potentially functional SNPs that might disturb the binding of transcription factors, 9 SNPs were excluded because of high linkage disequilibrium (LD) analyzed by (r^2^ > 0.8). As a result, 7 SNPs (rs3733846 in miR-143/145, rs12538588 in miR-183/96/182, rs1292060 in miR-21, rs763354 in miR-30a, rs9660710 in miR-200b/200a/429, rs12286521 in miR-210 and rs901975 in miR-451a) were genotyped in our study.

Genomic DNA was isolated from a leukocyte pellet by proteinase K digestion, followed by phenol-chloroform extraction and ethanol precipitation. All SNPs were genotyped by Illumina Infinium^®^ Human Exome BeadChip (Illumina Inc., San Diego, CA). All SNPs were successfully genotyped with call rates > 95%.

### *In silico* analysis

In order to evaluate whether promising SNPs harbor regulatory elements, we downloaded Chromatin Immunoprecipitation sequencing (ChIP-seq) and DNaseI Hypersensitivity (DNaseI HS) data in normal lung cell line (Normal Human Lung Fibroblasts, NHLF) and lung cancer cell line (A549) from Encyclopedia of DNA Elements (ENCODE) project (https://www.encodeproject.org/), and visualized these regulatory elements using Integrative Genomics Viewer (IGV) (http://www.broadinstitute.org/igv/). We also used the HaploReg resource V2 (http://compbio.mit.edu/HaploReg) to examine the risk loci and variants in high LD (r^2^ > 0.8 in Asian from the 1000 Genomes Project) for functional elements available from the Epigenome roadmap project [38].

To assess miRNA expression, level 3 miRNA isoform quantification data (reads per million of total reads mapping to a mature microRNA) in LUAD tumors and adjacent normal tissues were retrieved from TCGA portal on date 07/15/2014 (http://cancergenome.nih.gov/). We filtered out cross-mapped regions and then summed over the reads per million miRNAs mapped (RPM) values for each mature miRNA according to the previous study [37]. After normalization by using the EdgeR package [39], the expression data of interest miRNAs were extracted and were log2-transformed for further analysis.

In order to bridge the gap between the genotype and phenotype, we have applied for the Affymetrix SNP 6.0 Level 1 data of TCGA LUAD samples to extract genotypes of rs9660710 and rs763354. Because the chip didn't include the genotype information of rs9660710, we further imputed the genotypes of rs9660710 for TCGA samples by using IMPUTE2 (1000 Genomes Phase I integrated variant set b37 March 2012 release). The level 3 TCGA methylation data (Illumina's Infinium HumanMethylation450 Beadchip) for 32 LUAD normal tissues and paired 29 tumor tissues were downloaded from the TCGA data portal on date 07/15/2014.

### Statistical analysis

Differences between cases and controls in the distribution of demographic characteristics were evaluated by the χ^2^ test (for categorical variables) or Student's *t*-test (for continuous variables). Genotype frequencies in control group were tested for the Hardy-Weinberg equilibrium by using goodness-of-fit χ2 test. OR and their 95% CI were calculated by using logistic regression analysis to evaluate the associations between genotypes and lung cancer risk with adjustment for age, gender and smoking. For survival analysis, the distribution of demographic and clinical variables according to death status was tested by the χ^2^ test. Log-rank test was applied to compare the survival time in different subgroups categorized by patient characteristics and genotypes. Univariate and multivariate Cox proportional hazard regression analysis were performed to estimate the crude or adjusted hazard ratio (HR) and 95%CI with adjustment of age, gender, smoking, clinical stage, chemotherapy or radiotherapy, surgery status and histological types. The Chi-square-based Q test was applied to test the heterogeneity of associations between subgroups. The paired two-sample Student's *t*-test was used to compare the miRNA expression or methylation levels of CpG sites in paired tumor and adjacent normal tissues from TCGA dataset. Spearman's rank correlation was used to evaluate genotype-expression and genotype-methylation associations. All of the statistical analyses were performed with R software (version 3.2.2; The R Foundation for Statistical Computing). A two-sided *P* value of < 0.05 was considered statistically significant.

## SUPPLEMENTARY MATERIALS


