# Supplementary material for: Genetic variants in regulatory regions of microRNAs are associated with lung cancer risk
Source: Oncotarget. 2016 Jun 25;7(30):47966–74. doi: 10.18632/oncotarget.10299 (PMC5216992; doi:10.18632/oncotarget.10299)
Supplement: Supplementary file 1 [file oncotarget-07-47966-s001.pdf]

Genetic variants in regulatory regions of microRNAs are associated with lung cancer risk

Supplementary Materials

Supplementary Table S1: Characteristics of subjects

Variable	Cases (*N* = 1,341)	Controls (*N* = 1,982)	*P*
	N (%)	*N* (%)	
Age			
< 60	589 (43.9)	873 (44.0)	0.972
≥ 60	752 (56.1)	1109 (56.0)	
Gender			
Male	949 (70.8)	1358 (68.5)	0.179
Female	392 (29.2)	624 (31.5)	
Smoking			
Never	522 (38.9)	1020 (51.5)	< 0.001
Ever	819 (61.1)	962 (48.5)	
Histological types			
Squamous cell carcinoma	481 (35.9%)		
Adenocarcinoma	860 (64.1%)		

Supplementary Table S2: Patients' characteristics and clinical features in the survival analysis

Variables	Patients $N = 1001$	Deaths $N = 545$	MST (Months)	Log-rank P	HR (95% CI)
Age				0.903	
< 60	428 (42.8)	229	25.8		1.00
≥ 60	573 (57.2)	316	27.0		0.99 (0.83–1.17)
Gender				0.034	
Male	695 (69.4)	399	25.0		1.00
Female	306 (30.6)	146	32.9		0.81 (0.67–0.99)
Smoking				0.027	
Never	401 (40.1)	197	30.0		1.00
Ever	600 (59.9)	348	23.9		1.22 (1.02–1.45)
Surgical operation				< 0.001	
No	325 (32.5)	247	14.6		1.00
Yes	676 (67.5)	298	44.4		0.33 (0.27–0.39)
Clinical stage[a]				< 0.001	
I/II	417 (41.7)	165	59.3		1.00
III/IV	564 (56.3)	370	18.9		2.71 (2.25–3.27)
Histological types				0.060	
Squamous cell carcinoma	344 (34.4)	198	22.2		1.00
Adenocarcinoma	657 (65.6)	347	28.5		0.85 (0.71-1.01)
Chemotherapy or radiotherapy[b]				0.026	
No	236 (23.6)	110	30.8		1.00
Yes	757 (75.6)	430	25.6		1.27 (1.03–1.56)

[a]Clinical stage information was available in 981 NSCLC patients.

[b]Chemotherapy or radiotherapy information was available in 993 NSCLC patients.

Supplementary Table S3: Genotyping results of 7 SNPs and NSCLC survival

Gene	SNP	Allele[a]	Patients[b]	Deaths[b]	MST[b] (Months)	Log-rank *P*			
						Additive model	Dominant model	Codominant model[c]	
								het	hom
miR-143/145	rs3733846	A/G	457/428/115	257/223/65	25.6/29.4/21.6	0.353	0.436	0.256	0.585
miR-183/96/182	rs12538588	G/A	910/91/0	493/52/0	26.0/27.0/0.0	0.693	0.693	0.693	—
miR-21	rs1292060	A/G	348/463/190	192/248/105	25.8/27.4/25.6	0.699	0.934	0.699	0.579
miR-30a	rs763354	G/A	370/476/152	197/255/91	28.1/26.0/25.8	0.468	0.425	0.657	0.204
miR-200b/200a/429	rs9660710	C/A	310/504/187	165/266/114	27.4/28.5/22.3	0.169	0.571	0.975	0.101
miR-210	rs12286521	A/G	550/395/56	301/216/28	26.0/25.8/27.2	0.913	0.812	0.891	0.672
miR-451a	rs901975	G/A	425/456/120	225/259/61	25.8/24.9/32.3	0.244	0.986	0.592	0.187

[a]Major/minor allele.

[b]Major homozygote/heterozygote/minor homozygote.

[c]het: heterozygote versus major homozygote; hom: minor homozygote versus major homozygote.

Supplementary Table S4: Associations of 7 SNPs with NSCLC prognosis

Genotype	Patients	Deaths	MST (Months)	Crude HR (95% CI)	Adjusted HR (95% CI)[a]	P^a
rs3733846						
AA	457	257	25.6	1.00	1.00	—
AG	428	223	29.4	0.90 (0.75–1.08)	0.92 (0.76–1.10)	0.356
GG	115	65	21.6	1.07 (0.82–1.41)	0.99 (0.75–1.31)	0.952
AG/GG	543	288	28.1	0.94 (0.79–1.11)	0.94 (0.79–1.11)	0.420
Additive model	—	—	—	0.99 (0.87–1.12)	0.97 (0.85–1.10)	0.639
rs12538588						
GG	910	493	26.0	1.00	1.00	—
GA	91	52	27.0	1.06 (0.80–1.41)	1.02 (0.76–1.36)	0.910
AA	0	0	0	—	—	—
GA/AA	91	52	27.0	1.06 (0.80–1.41)	1.02 (0.76–1.36)	0.910
Additive model	—	—	—	1.06 (0.80–1.41)	1.02 (0.76–1.36)	0.910
rs1292060						
AA	384	192	25.8	1.00	1.00	—
AG	463	248	27.4	0.97 (0.80–1.17)	1.00 (0.82–1.21)	0.993
GG	190	105	25.6	1.07 (0.84–1.35)	0.96 (0.76–1.23)	0.765
AG/GG	653	353	26.5	0.99 (0.83–1.18)	0.99 (0.83–1.18)	0.897
Additive model	—	—	—	1.02 (0.91–1.15)	0.98 (0.87–1.11)	0.791
rs763354						
GG	370	197	28.1	1.00	1.00	—
GA	476	255	26.0	1.17 (0.91–1.50)	1.09 (0.90–1.32)	0.380
AA	152	91	25.8	1.04 (0.87–1.26)	1.26 (0.98–1.62)	0.076
GA/AA	628	346	25.8	1.07 (0.90–1.28)	1.13 (0.94–1.35)	0.186
Additive model	—	—	—	1.07 (0.95–1.21)	1.12 (0.99–1.26)	0.083
rs9660710						
CC	310	165	27.4	1.00	1.00	—
CA	504	266	28.5	1.00 (0.82–1.21)	1.08 (0.89–1.32)	0.443
AA	187	114	22.3	1.22 (0.96–1.55)	1.24 (0.97–1.58)	0.088
CA/AA	691	380	25.8	1.05 (0.88–1.27)	1.12 (0.93–1.35)	0.223
Additive model	—	—	—	1.09 (0.97–1.23)	1.11 (0.98–1.25)	0.095
rs12286521						
AA	550	301	26.0	1.00	1.00	—
AG	395	216	25.8	0.99 (0.83–1.18)	1.07 (0.89–1.28)	0.476
GG	56	28	27.2	0.92 (0.62–1.36)	0.97 (0.66–1.43)	0.881
AG/GG	451	244	25.8	0.98 (0.83–1.16)	1.05 (0.89–1.25)	0.544
Additive model	—	—	—	0.98 (0.85–1.12)	1.03 (0.89– 1.19)	0.697
rs901975						
GG	425	225	25.8	1.00	1.00	—
GA	456	259	24.9	1.05 (0.88–1.26)	1.01 (0.84–1.21)	0.924
AA	120	61	32.3	0.83 (0.62–1.10)	0.91 (0.68–1.21)	0.498
GA/AA	576	320	26.5	1.00 (0.84–1.18)	0.99 (0.83–1.18)	0.888
Additive model	—	—	—	0.95 (0.84– 1.08)	0.97 (0.85–1.10)	0.635

MST, median survival time; HR, hazard ratio; CI, confidence interval.

[a]Adjusted for age, gender, smoking, clinical stage, chemotherapy or radiotherapy, surgery and histological types.

Supplementary Table S5: Summary of genomic annotation by HaploReg for SNPs in high LD with rs9660710 ($r^2 > 0.80$)

Variant	LD (r^2)	Ref	Alt	ASN freq	Promoter histone marks	Enhancer histone marks	DNAse	Proteins bound	Motifs changed	RefSeq genes
rs6604973	0.82	G	A	0.57	ST.SMUS28, R.MUC29, R.SMUS	10 cell types	—	—	—	7.1kb 5′ of MIR200B
rs1539636	0.90	T	C	0.59	COL. MUC32, R.MUC31	8 cell types	Medullo, SK-N-SH_RA	—	8 altered motifs	5.6kb 5′ of MIR200B
rs1539634	0.90	C	T	0.59	COL. MUC32, R.MUC31	9 cell types	—	—	SP1,SRF	5.4kb 5′ of MIR200B
rs9442384	0.90	T	C	0.59	4 cell types	9 cell types	Osteobl	—	Sin3Ak-20	5.2kb 5′ of MIR200B
rs9442385	0.90	T	G	0.59	4 cell types	9 cell types	Osteobl	—	10 altered motifs	5.1kb 5′ of MIR200B
rs145121017	0.90	CCCCA	C	0.59	5 cell types	15 cell types	Fibrobl	—	34 altered motifs	5.1kb 5′ of MIR200B
rs9660710	1.00	A	C	0.58	18 cell types	9 cell types	LNCaP	—	BRCA1, CHD2	3.1kb 5′ of MIR200B
rs1891905	0.85	C	T	0.61	14 cell types	14 cell types	Fibrobl, Caco-2, H7-hESC	4 bound proteins	—	2.3kb 5′ of MIR200B
rs1891904	0.82	C	A	0.60	14 cell types	14 cell types	16 cell types	5 bound proteins	CTCF,YY1	2.2kb 5′ of MIR200B
rs7549819	0.80	C	T	0.60	13 cell types	19 cell types	5 cell types	USF2	4 altered motifs	1.5kb 5′ of MIR200B
rs7518873	0.81	T	C	0.58	PFF.2	13 cell types	GM12891	—	6 altered motifs	209bp 3′ of MIR200A
rs36015232	0.83	C	9-mer	0.59	—	14 cell types	LNCaP, Osteobl	—	—	358bp 3′ of MIR200A
rs7521584	0.86	T	A,G	0.59	—	15 cell types	HSMMtube, Chorion, GM19240	—	—	425bp 5′ of MIR429

The LD information was derived from the 1000 Genomes Project ASN (East Asian) data for the rs9660710 (marked in bold) and its surrogates. DNAse, Proteins bound and Motifs changed demonstrated evidence of Dnase hypersensitivity sites or transcription factor occupancy as shown by the HaploReg v2 analysis.

Supplementary Table S6: Summary of genomic annotation by HaploReg for SNPs with rs763354 in high LD (r² > 0.80)

Variant	LD (r^2)	Ref	Alt	ASN freq	Promoter histone marks	Enhancer histone marks	DNAse	Proteins bound	Motifs changed	RefSeq genes
rs9351810	0.95	C	T	0.39	4 cell types	30 cell types	7 cell types	CFOS, CJUN, GATA2	Dbx1,Osr	9.8kb 3′ of MIR30A
rs9364165	0.96	C	T	0.39	MSC.ADIPC, ST.SMUS28	29 cell types	—	5 bound proteins	13 altered motifs	9.1kb 3′ of MIR30A
rs9364166	0.96	C	T	0.38	—	24 cell types	—	—	HLF	6.6kb 3′ of MIR30A
rs4291065	0.86	T	C	0.41	—	8 cell types	—	—	NRSF	6.2kb 3′ of MIR30A
rs9364167	0.96	G	A	0.38	—	6 cell types	—	—	5 altered motifs	5.6kb 3′ of MIR30A
rs9351811	0.99	G	A	0.38	—	4 cell types	—	—	4 altered motifs	4.4kb 3′ of MIR30A
rs1405848	0.90	C	T	0.41	—	6 cell types	—	—	5 altered motifs	3.9kb 3′ of MIR30A
rs2222722	0.89	G	A	0.41	9 cell types	39 cell types	HCFaa, HMVEC-dLy-Ad	CEBPB	Rhox11	387bp 5′ of MIR30A
rs763354	1.00	C	T	0.39	—	25 cell types	—	—	AIRE,Sox	2.3kb 5′ of MIR30A
rs9283841	0.90	T	C	0.41	—	20 cell types	—	—	NF-I	2.8kb 5′ of MIR30A
rs7349905	1.00	G	C	0.39	—	28 cell types	HRCEpiC, HRE,PANC-1	—	AP-1,Mxi1, SREBP	5.4kb 5′ of MIR30A
rs9342836	1.00	T	C	0.39	ST.SMUS28, KID.FE, LIV.A	13 cell types	HA-sp	—	11 altered motifs	2.7kb 3′ of C6orf155
rs6902769	0.91	T	C	0.41	7 cell types	20 cell types	—	—	4 altered motifs	1.5kb 3′ of C6orf155
rs1852578	0.90	T	A,G	0.41	KID.FE, LIV.A, SK.MUS62	14 cell types	—	—	—	705bp 3′ of C6orf155
rs1918478	1.00	G	T	0.39	KID.FE, LIV.A, SK.MUS62	15 cell types	—	—	GATA,LUN-1	75bp 3′ of C6orf155
rs6455418	0.86	A	T	0.40	—	17 cell types	—	—	ZID	C6orf155
rs113733589	0.86	G	A	0.37	79 cell types	CC.TPC	H1-hESC, Chorion,iPS	CTCF,CFOS, HAE2F1	8 altered motifs	C6orf155
rs12214632	0.81	T	A	0.40	—	—	—	—	CDP,Mef2	2.6kb 5′ of C6orf155
rs12192535	0.81	G	C	0.40	—	—	—	—	5 altered motifs	2.7kb 5′ of C6orf155
rs9364168	0.89	A	G	0.37	—	—	—	—	25 altered motifs	4kb 5′ of C6orf155
rs9346470	0.82	T	C	0.38	—	—	—	—	DMRT1, Dobox4	5.9kb 5′ of C6orf155

The LD information was derived from the 1000 Genomes Project ASN (East Asian) data for the rs763354 (marked in bold) and its surrogates. DNAse, Proteins bound and Motifs changed demonstrated evidence of Dnase hypersensitivity sites or transcription factor occupancy as shown by the HaploReg v2 analysis.
